# Tropical forest cover, oil palm plantations, and precipitation drive flooding events in Aceh, Indonesia, and hit the poorest people hardest

**DOI:** 10.1371/journal.pone.0311759

**Published:** 2024-10-14

**Authors:** Muhammad Irfansyah Lubis, Matthew Linkie, Janice Ser Huay Lee

**Affiliations:** 1 Asian School of the Environment, Earth Observatory Singapore, Nanyang Technological University, Singapore, Singapore; 2 Wildlife Conservation Society, New York, New York, United States of America; Science Hub Nepal, NEPAL

## Abstract

Tropical forest loss and degradation in watersheds disrupt essential ecosystem services that regulate water flow, often causing devastating floods that impact agricultural productivity and impoverish downstream communities. Despite its importance, evaluations of the interconnectedness between the depletion of hydrological services and flooding lack an evidence-base in the Global South and, therefore, have little influence on policy dialogue. In this study, we focus on the forest-rich province of Aceh, Indonesia, using local and national newspaper articles to compile information on flood events between 2011 and 2018. We explored spatio-temporal flood patterns with a combination of climatic, topographic, and environmental factors. We compiled 2,029 reported flood events in mainland Aceh located in 20 of the 21 districts/cities, with a disproportionately high occurrence (71%) in four districts. The trend of flood events exhibited an increasing pattern between 2011 and 2018. Over this period, floods displaced ~158,000 people and damaged ~24,500 houses and ~11,500 ha of agricultural land. Our generalized linear mixed-effect model found that reported flood events were more likely to occur in areas with lower tree cover, more oil palm plantations, and higher precipitation. Areas with a lower human population density and higher poverty rates were found to be most susceptible to flooding events. Our findings highlight the critical link between forest preservation and flood prevention, and the irreplaceable role that forests play in ensuring the well-being of local communities, especially those affected by poverty. Our study underscores the importance of considering these interconnected factors in future land use and economic development plans and policies.

## Introduction

The degradation of tropical forest ecosystems threatens the health and safety of human populations. Changes in the structure and function of these tropical ecosystems can yield undesirable consequences, such as zoonotic disease emergence, increased human-wildlife conflicts, and greater landslide risk [1–4]. Tropical forest degradation over the past decades has disrupted and diminished a range of essential ecosystem services, in particular watershed services that regulate ground and surface water flow, control soil erosion, and stabilize riverbanks [5–8]. Alterations to these hydrological processes, particularly from forest loss and degradation, often leads to increased frequency and severity of flooding events [9–11].

There are several mechanisms through which tropical forests mitigate flooding, notably evapotranspiration, water infiltration to the soil, and water retention of infiltrated water [12–14]. Tropical forest soils tend to have high hydraulic conductivity due to a dense tree root network and soil organisms that break up the soil [12]. This allows for greater water storage than other land use types, especially agricultural or urban areas that typical replace standing forest [8, 11]. Water infiltration also prevents soil erosion and, consequently, reduces the amount of sediment entering rivers, thereby reducing river bank overflow [7, 12].

While the importance of forest ecosystems in mitigating flooding is generally well understood [10, 15], quantifying the relationship between tropical forest cover decline and flood events is complex because it is rarely linear and measuring hydrological functions are difficult under field conditions [16]. Previous studies on the global and regional associations between forest cover loss and flooding incidents have, therefore, tended to rely on secondary data sources.

Bradshaw et al. [9] found a significant and positive correlation between the frequency of large floods in 56 developing countries and forest loss that occurred from 1990 to 2000. This dataset was reanalyzed with the inclusion of country level data for human population density and found to provide a stronger explanation than forest cover change alone [14]. A study of 31 river basins in Peninsular Malaysia found that the replacement of tropical forests with oil palm and rubber plantations from 1984 to 2000 significantly increased the number of days flooded during the wettest months of the year [17]. These studies highlight the need for a greater understanding of a complex process through studying regional effects of forest cover loss and flooding in the Global South. Yet, these studies are generally lacking because detailed records of flood events are scattered across data sources [18, 19]. To overcome this data deficiency, several studies have used alternative approaches, such as newspaper and social media reports.

When a flood event occurs, it is typically covered by local and national media through their network of site-based journalists [20]. In the absence of comprehensive government datasets, newspaper reports provide valuable near-real time information for studying flood occurrence, risk, and hazards. For example, Yagoub et al., [21] found a strong association between flood prone areas that were generated from spatial analyses incorporating expert judgement and those derived from newspaper reports that covered the same locations and period. Tellman et al., [22] found that news reports had several advantages over satellite-based observations in monitoring floods, such as in areas with persistent cloud cover, complex terrain, dense forest cover, and small or flash floods. Wells et al., [23] compared interviews with village leaders and newspaper reports of flooding events in Borneo and found strong spatial association. To address possible data limitations on flood reporting by governments in countries in the Global South, new tools such as data mining from social media [19, 24, 25] and newspaper reports [9, 20, 23] have been developed and applied to fill this knowledge gap. The use of social and online media data mining, while relatively new, is playing an increasingly important role in conservation science, such as in monitoring illegal wildlife trade, mitigation of human and wildlife conflict, and mapping ecosystem services [26–28].

In our study, we investigate the underlying drivers of flood events in the Indonesian province of Aceh. Aceh contains the largest area of intact forest on Sumatra, the sixth largest island in the world, but forest cover is threatened by illegal logging, conversion to smallholder farmland, and infrastructure development [29–33]. Forest loss in Aceh has anecdotally been linked to flooding events [34–37], yet hitherto untested. To investigate this association, we constructed a comprehensive village-level flood event database for mainland Aceh province for the years 2011 to 2018 using online newspaper sources. Our study aimed to: 1) Map and quantify the spatial and temporal patterns of reported flood events and their socio-economic impacts; 2) Assess the direct factors that drive reported flood events; and from this, 3) Identify at-risk areas of reported flood occurrence and explore its association with human population density and poverty data layers to guide future spatial, economic and infrastructure development planning.

## Materials and methods

### The ecological significance of Aceh’s forest

Aceh Province located in the Indonesian island of Sumatra, is renowned for its vast tropical rainforests, including the globally significant Leuser Ecosystem. This intact forest ecosystem is a biodiversity hotspot, providing a habitat for endangered species like Sumatran orangutans, tigers, elephants, and rhinoceros [38]. The topography of Aceh ranges from sea level to a rugged interior that ascends to 3,466 meters, the peak of Mount Leuser, and gives rise to the following forest types ‐ peat swamp (0 m asl), lowland (0–300 m asl), hill (300–800 m asl), sub-montane (800–1,300 m asl), montane (1,300–1,800 m asl), upper-montane (1,800–2,500), and tropical alpine (> 2,500 m asl; [39]).

Peatland forests in Aceh hold great importance due to their unique characteristics and ecological functions, such as carbon sequestration, coastal flood control, and fisheries management [40]. These forests even played a role in mitigating the impact of the 2004 Indian Ocean tsunami on coastal communities [41]. At higher elevations, tropical moist montane forest and tropical high altitude shrub land capture and gradually release rainwater to the lowlands, providing a year-round supply of water [42]. This ecosystem service is vital for irrigating rice fields and tree crops, such as oil palm, and for controlling excess run-off, which is important considering that Aceh receives a high annual average rainfall of 1,500–2,500 mm [43]. The ecosystem services provided by Aceh’s forests to agriculture have an annual estimated economic value of USD 206 million based on a forest conservation scenario [44].

Aceh’s land use has undergone three distinct changes. In 1971, forest covered 75% of the province, with only 10% allocated for cultivation [45]. Selective logging in private sector concessions occurred from 1979 to 1990, which was followed by illegal logging and agricultural encroachment into primary forest [44]. Although the timber boom declined in the 1990s, large-scale timber activities and mining operations continued. Deforestation averaged 30,952 hectares per year from 1990 to 2000 [46]. After the Helsinki Peace Accord was signed in 2005, legal and illegal timber operations were scaled-up to assist the province’s post-tsunami reconstruction efforts [31] and swiftly followed by a province-wide logging moratorium in 2007 and oil palm moratorium in 2011 [47, 48].

### Compiling reported flood events from online articles

**Fig 1:**
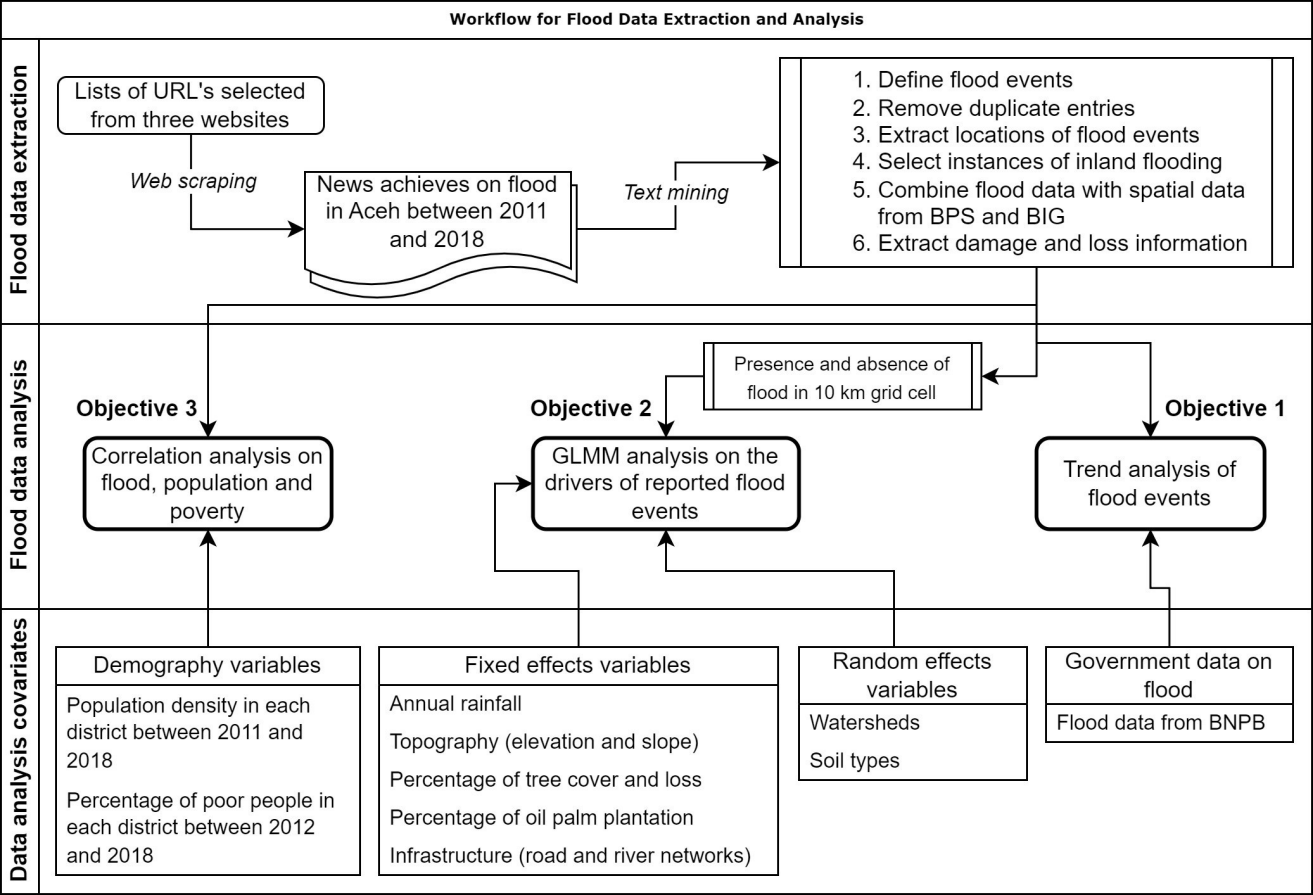
Flow chart illustrating the process for compiling and analyzing the reported flood events in Aceh province.

We compiled flood event reports from the most comprehensive news archives in Indonesia and Aceh, namely the online data libraries of Aceh Tribune News (http://aceh.tribunnews.com), Kompas (http://regional.kompas.com), and Antara News (http://aceh.antaranews.com). By using relevant Indonesian keywords such as "*banjir*" (flood), "Aceh," and "*tahun*" (year), we conducted searches on each newspaper’s website. The combinations of these keywords yielded a varying number of flood events reports, ranging from 11 to 82 reports for the different study years. On average, the local newspaper, Tribun News, provided a greater number of reports compared to the national newspapers, Kompas and Antara. Due to the limited availability of flood information before 2011, as Antara News did not exist, we restricted our search to the years between 2011 and 2018. This search process resulted in the compilation of 1,003 online articles documenting flooding events during this period. The URLs of all the online articles were copied into a spreadsheet for the web scraping process. To ensure data quality, we used an automated process to remove duplicate articles and those that lacked flood location information at the village-level, which was the smallest administrative unit used in our analysis (Fig 1).

We used the term "reported flood events" to avoid any misinterpretation [49]. In our context, a reported flood event refers to a flood event that took place in a specific village on any date within a month. For example, if two or more events happened in the same village, but were reported by different news agencies or the same agency for different dates during the same month, we considered this as a single event to avoid possible duplication and over-estimation. Where a single reported flood event occurred in multiple villages within the same month, this would be recorded as multiple events in our dataset since our analysis focused on the village level. This process removed 470 articles, leaving 533 independent articles for subsequent analysis. We developed an R script to automate the extraction of reported flood information (date, title, body text of articles, and location of flooding down to the village level). For determining the location of flood events, we used a three-step text mining process, spatialized the data using administrative boundaries provided by the Indonesian Statistics Bureaus (BPS) and extracted the village points using data from the Indonesian Geospatial Agency (BIG; see S1 Text for more details on this process).

Flooding events are generally classified as being either a coastal flood, fluvial flood, or pluvial flood [50]. Coastal floods occur when seawater inundates land along the coast, usually resulting from a combination of high tide, heavy rain, and onshore winds [51]. Fluvial or riverine floods occur when a river overflows its banks due to intense precipitation. Pluvial floods occur when rainwater accumulates beyond the soil’s capacity to absorb it, even in areas located far from water bodies. To maintain the focus of our analysis on inland flooding and exclude coastal flood data, we used a high-resolution digital elevation model (DEM) and removed flood events below 1 meter [52, 53]. We therefore aimed to explore the association between flooding and various environmental variables within an inland context (Fig 1).

The severity of reported flood impacts was manually recorded. In cases where multiple sources provided information on flood severity impacts, such as the number of people killed, injured, missing, or displaced, property damage, and agricultural land damage, we extracted the upper metric reported by a news agency.

All data, including the flood information and georeferencing process, were downloaded and processed using R software version 4.0.2. Several R packages were used for different tasks, including the "rvest" package for extracting information from websites [54], the "stringr" package for conducting text mining [55], the "tidyverse" package for cleaning and reformatting the dataset [56], and the "sp," "rgdal," and "rgeos" packages for georeferencing and handling spatial data [57–59].

### Government data on flood events and their impacts

Relying solely on news articles for flood events may introduce bias, as densely populated areas, for example, may receive greater media attention and lead to overrepresentation [14]. A comprehensive understanding requires considering scientific studies, government reports, and local community records for a balanced assessment of flood events [19, 23]. To address this, we compared reported flood events and their impacts with the Indonesian Disaster Management Agency dataset (BNPB: https://gis.bnpb.go.id/). The BNPB is the official government source that provides historical data on various types of disasters, including earthquakes, tsunamis, floods, landslides, and volcanic eruptions, along with their societal impacts down to the district level.

### Direct drivers of flood events in the tropics

In tropical regions, floods may be influenced by various direct drivers, including rainfall, topography, forest cover and changes, and infrastructure development. Intense and frequent rainfall events, often associated with monsoonal systems and climate variability, contribute significantly to flooding [60, 61]. The tropical region’s topography, characterized by steep slopes and narrow valleys, can intensify runoff during heavy rainfall [61, 62]. Forest cover plays an important role in reducing the frequency of flooding, although it may not affect larger flood events [13, 63]. Changes in forest cover, such as deforestation for agriculture and urbanization, alter the hydrological cycle, leading to increased surface runoff and reduced infiltration capacity [11, 23, 64, 65]. The expansion of oil palm plantations, particularly in peatlands, is predicted to heighten land susceptibility to flooding [66]. Furthermore, infrastructure development, such as dams, river channelization, and inadequate water management practices, can contribute to flood events [11, 67].

To investigate the direct influence of these factors on reported flood events in Aceh, we used the following variables. Annual rainfall (mm/year) data for Aceh from 2011 to 2018 were obtained from the Climate Hazards Group InfraRed Precipitation with Station data (CHIRPS) at a 1 km resolution [68]. Topography characteristics, including elevation (m asl) and slope (degree), were derived from a 30 m resolution digital elevation model [69]. Percentage area of tree cover and tree cover loss were obtained from the global forest change dataset version 1.7 [70] (see S2 Text for more information on processing tree cover and tree cover loss). The extent of oil palm plantations up to 2017 was obtained from Danylo et al., [71]. Infrastructure data, including roads and river channelization were obtained from the Indonesian Geospatial Agency at scale 1:50,000 [72]. These variables (S1 Table and Fig 1) were extracted at 10 km grid cell using ArcMap v10.4.1 and R program [73].

## Data analysis

### Trend analysis of flood events

To conduct a comprehensive analysis of flood events and their socio-economic impacts, we carried out a temporal and spatial assessment using both our own flood event data and data provided by the Indonesian Disaster Management Agency (BNPB; https://gis.bnpb.go.id/) for the years 2011 to 2018 in Aceh Province. The annual pattern was assessed (Mann-Kendall test) in R using ‘Kendall” package [74].

### GLMM analysis on the drivers of reported flood events

To analyze the relationship between reported flood events and various environmental variables in Aceh, we used a two-step regression approach. We first focused on selecting the most appropriate regression model that suits our data. The dependent variable, representing the presence or absence of reported floods, was extracted from reported flood event data obtained from online articles. We used 10 km grid cells as the unit of analysis, where each grid cell was assigned a value of 1 to indicate the presence of a reported flood event and 0 to indicate its absence. This approach was chosen instead of using the exact count of reported floods from each grid cell to mitigate potential bias stemming from our specific flood definition. The choice of a 10 km grid cell size was determined as it aligns with the smallest village size in mainland Aceh, considering that village boundaries often vary considerably in shape and size. Simultaneously, we extracted the corresponding independent variables at the same grid cell level (S1 Table and Fig 1).

To identify the optimal regression model for our dataset, we first compared two models: the Generalized Linear Model (GLM) and the Generalized Linear Mixed Model (GLMM). We considered watershed and soil types as random effects in the GLMM models (S2 Table and Fig 1) since these factors might influence the variability of flood events in Aceh. Each model included only two uncorrelated variables: annual rainfall and percentage of tree cover. Using the Akaike Information Criterion corrected for small sample sizes (AICc; [75]), we ranked the models, leading us to select the GLMM with watershed as a random effect as the most suitable model for our reported flood datasets (S2 Table).

The watershed polygons were obtained from the Indonesian Geospatial Agency. These were delineated using GIS software and were derived from various data sources, including a Digital Elevation Model (DEM) at a resolution of 90 m, a toponymy (place names) map at a resolution of 90 m, and remote sensing imagery at a resolution of 30 m [76]. Additionally, the soil types were derived from the digital soil map of the world published by FAO/UNESCO [77].

In the second step, we employed GLMM models, incorporating watershed as a random effect, and eight environmental variables such as annual rainfall, elevation, slope, percentage of tree cover, percentage of tree cover loss, percentage of oil palm, and infrastructure development (road and river networks) as fixed effects (Fig 1). Furthermore, we included the year as an additional covariate to investigate the temporal trend of flood events in Aceh Province. To ensure robustness and minimize bias, we developed 15 candidate models (S3 Table) for exploring the relationship between flood events and environmental variables. Only variables with correlation coefficients less than ±0.6 were included in the models to avoid potential multicollinearity issues that could affect model accuracy (S1 Fig).

We evaluated the contribution of each variable to the model by comparing the magnitude of their respective beta coefficients and associated p-value. For instance, if the beta coefficient for percentage of tree cover in one model is greater than that for annual rainfall in another model, it suggests that, in the context of our analysis, percentage of tree cover has a stronger influence on reported flood events than annual rainfall (see S4 Table). To perform this assessment, we constructed three separate models with variables obtained from the top-ranked model from S3 Table, each incorporating a single variable (e.g., percentage of tree cover, percentage of oil palm, and annual rainfall), and then compared their respective beta coefficients.

The GLMM analysis was performed using the ’lme4’ package [78] in R. Model parsimony was evaluated using the AICc [75]. Additionally, we report both the conditional and marginal R-squared values [79] to quantify the proportion of variance explained by the fixed effects (Mar. R^2^) and both fixed and random effects (Cond. R^2^) in the model. Furthermore, the Intraclass Correlation Coefficient (ICC; [80]) was computed to assess the proportion of variance in the variable attributable to the variance between groups (i.e., random effects). The estimates from the top-ranked model and the comparisons between models, each including individual covariates were tabulated using the ’sjPlot’ package [81]. These comparisons aimed to assess the specific contribution of each covariate to the reported flood events.

To ensure the model validity and reliability from the selected top-ranked model, we performed a residual analysis, including diagnostic plot and spatial autocorrelation tests. We used R package DHARMa [82] with 1,000 iterations to examine the distribution of residuals and identify any patterns or systematic deviations from the assumed model structure. To assess the presence of spatial patterns in the residuals, we utilized Moran’s I test [83] using an ‘spdep’ package [57].

### Correlation analysis between reported flood events, human population, and poverty

We conducted an analysis to investigate the socio-economic impacts of flooding in Aceh, specifically examining its correlation with human population density and poverty rates at the district level (Fig 1). These variables were included in the correlation analysis to comprehend the impact of floods on the population, particularly those vulnerable due to poverty. In this study, poverty is defined as the number of individuals living below the poverty line in a district, with "poor individuals" being those whose average per capita monthly expenditure falls below the poverty threshold [84].

We conducted a comparative analysis between the total reported flood events and both the population density and the percentage of people living below the poverty line for each district from 2011 to 2018. Population density and poverty statistics were obtained from https://aceh.bps.go.id/, and we standardized the values using the "scale" function in R before the analysis. To explore these relationships, we performed Spearman’s rank correlation between reported flood events and both the population density and the percentage of people living below the poverty line.

## Result

### Spatial and temporal patterns of floods and its socio-economic impacts

**Fig 2:**
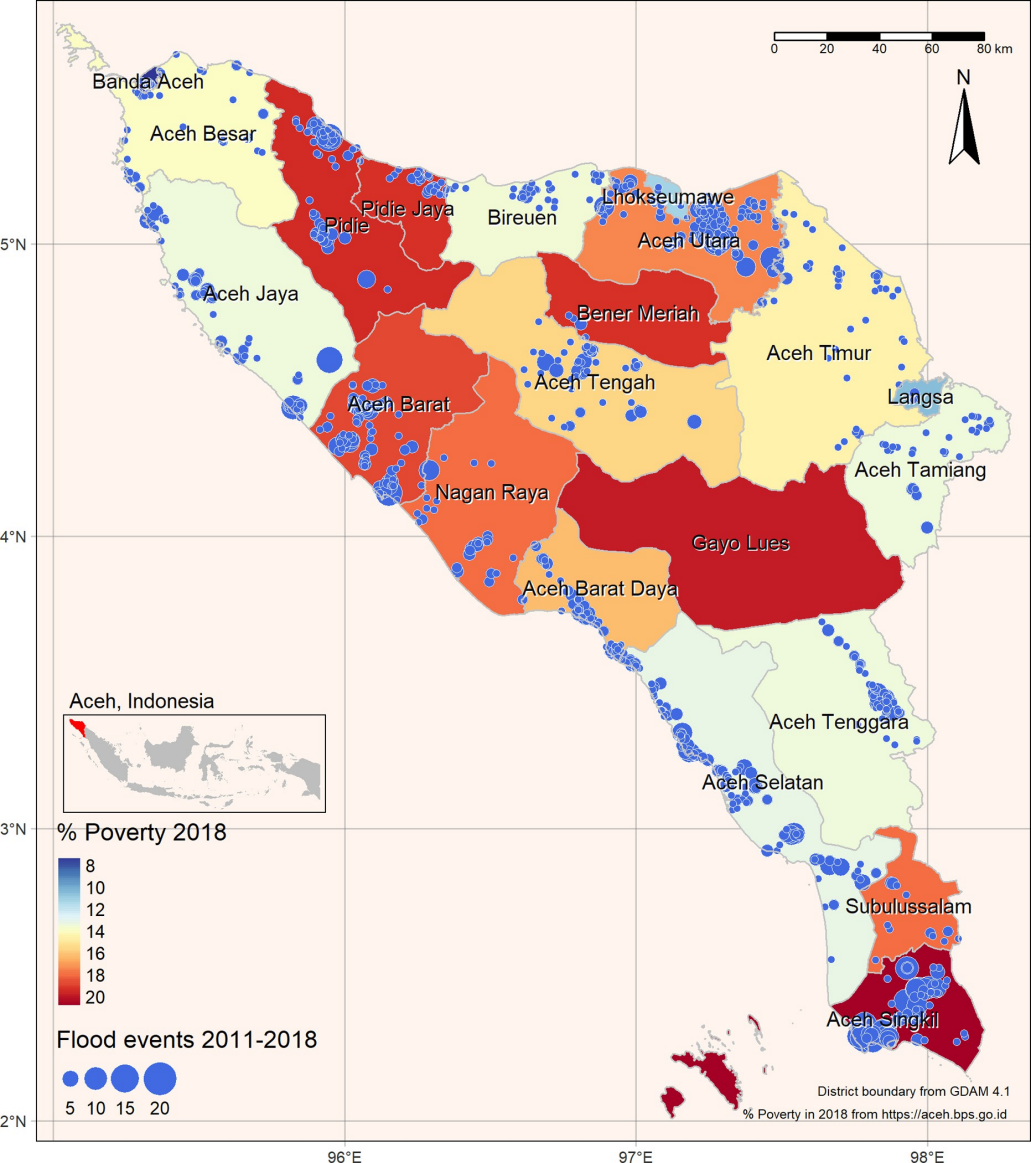
Spatial distribution of reported flood events between 2011 and 2018, and the percentage of poverty for each district in 2018 in Aceh Province.

Between 2011 and 2018, a total of 2,029 flooding events in Aceh province were extracted from 1,003 online articles (Fig 2). These floods occurred in 20 out of the 21 districts/cities and affected 985 out of the 5,353 villages on the mainland. They revealed that 71% of all flooding events took place in four districts: Aceh Utara (n = 426), Aceh Singkil (n = 290), Aceh Selatan (n = 269), and Aceh Barat (n = 221).

Amongst villages, the most severely affected were Pante Pirak (n = 16), Ujung Bawang (n = 14), Arongan (n = 13), and Meurebo (n = 13). These villages experienced an average of 1.75 floods per year, with a rising trend from 0.5 times in 2011 to 2.5 times in 2018. This trend indicates an almost six-fold increase compared to the provincial average for villages, which was 0.25 floods per year.

In comparison to the BNPB, which provided information at the district level, a total of 386 floods were recorded between 2011 and 2018, affecting 20 out of the 21 districts/cities. The districts with the highest number of flood events according to the BNPB dataset were Aceh Selatan (n = 40), Aceh Tenggara (n = 38), and Aceh Utara (n = 32).

**Fig 3:**
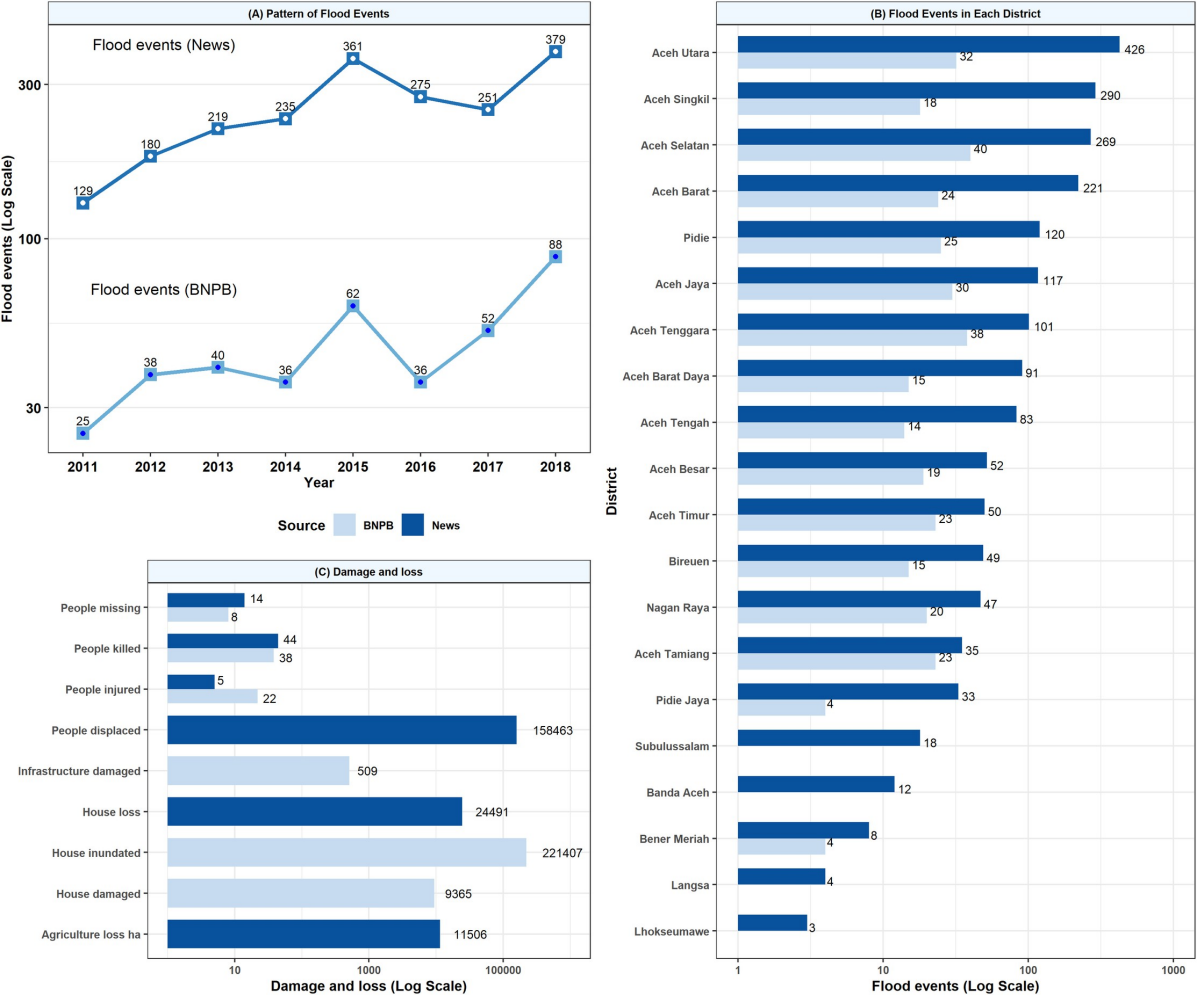
Summary statistics of flood events and their impacts in Aceh Province, including (A) the trend of annual flood events, (B) flood events in each district, and (C) damage and loss from flood events based on data from online articles and BNPB.

Despite the differences in the definition of flood events between the online news data (Mann-Kendall τ = 0.78, p < 0.01) and the government dataset (Mann-Kendall τ = 0.54, p < 0.1), both sources indicated an annual increase in flooding events (Fig 3A). Additionally, Aceh Utara and Aceh Selatan were identified as districts with high flood events in both datasets, providing further confirmation (Fig 3B).

When comparing the socio-economic impacts of floods between the online news data and the government dataset, some differences were observed, particularly in the number of people affected. The online news data, compared to the government dataset, reported slightly higher in numbers of people killed (44 vs. 38), injured (5 vs. 22), and missing (14 vs. 8) (Fig 3C).

### Drivers of flooding in Aceh province

**Fig 4:**
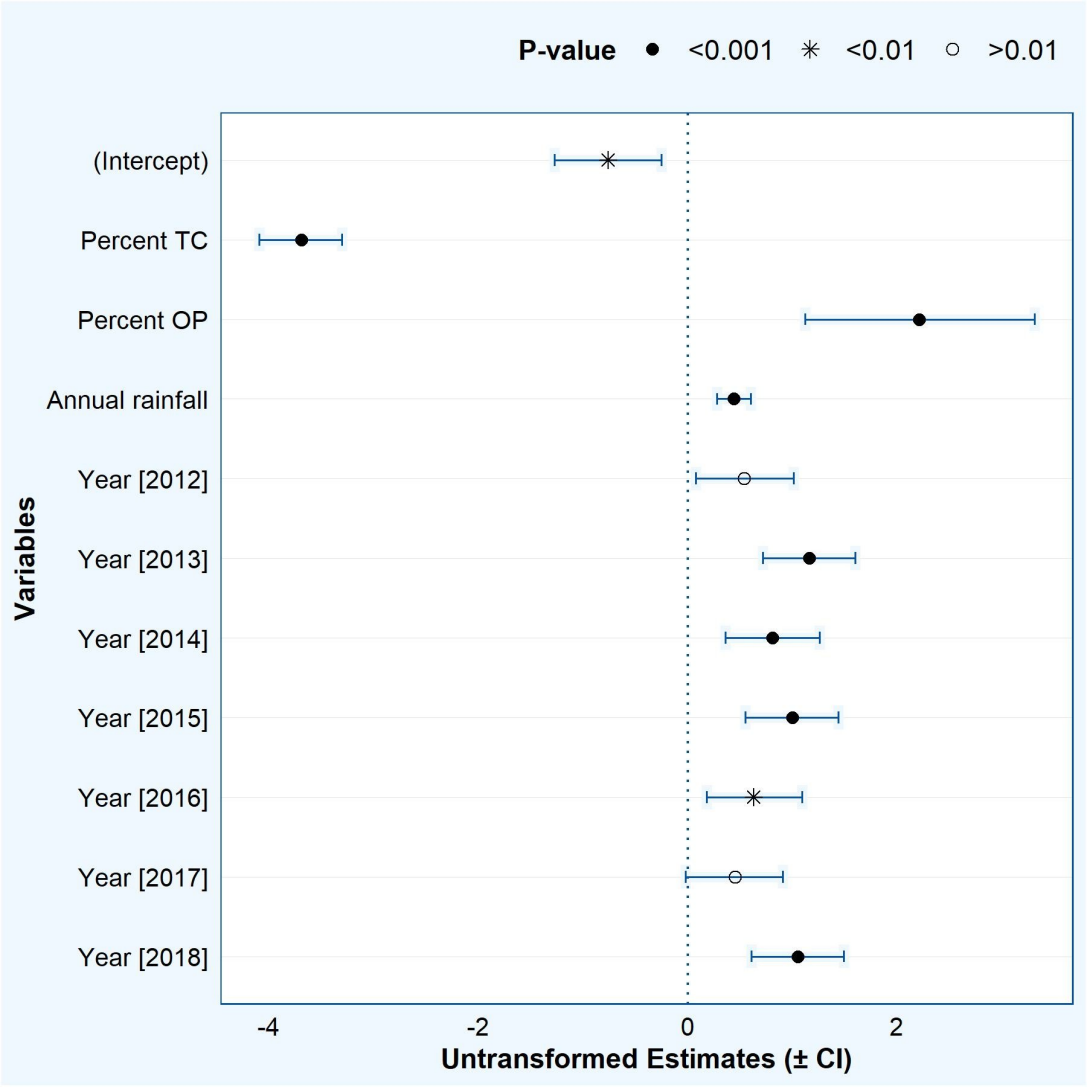
Untransformed regression estimate (± Confidence Interval) plots for the top-ranked candidate model for each year from 2011 to 2018.

Our top-ranked regression model (S3 Table) identified significant effects from several variables on reported flood events (S5 Table and Fig 4). The fixed effects in this top-ranked model explained 28% of the variability in these reports (Mar. R^2^). With the inclusion of watershed as a random effect, the variance explained by the model increased to 46% (Con. R^2^; S5 Table).

Our study found that a higher percentage of oil palm plantation and annual rainfall were significantly correlated with increased odds of floods (Untransformed Estimates (β) = 2.21, p < 0.001 and (β) = 0.44, p < 0.001, respectively), whereas a higher percentage of tree cover was linked to reduced odds of floods (β = -3.69, p < 0.001; Fig 4). The contribution of tree cover to reported flood events was the most significant (Mar. R^2^ = 0.24) of all variables tested, followed by the percentage of oil palm (Mar. R^2^ = 0.08), and annual rainfall (Mar. R^2^ = 0.02; S5 Table). The untransformed estimates for years indicated a significant increasing pattern of reported flood events for specific years, including 2013 (β = 1.16, p < 0.001), 2014 (β = 0.81, p < 0.001), 2015 (β = 1.00, p < 0.001), and 2018 (β = 1.05, p < 0.001; S5 Table and Fig 4).

The residual diagnostic test from the top-ranked model, as demonstrated in the qq plot (S2 Fig), indicates that the residuals show no substantial deviation from the straight line, indicating a good fit of the binomial mixed-effects model to the data. The spatial autocorrelation, although significant, in the residuals was small and therefore considered to have a negligible effect (Moran’s I statistic = 0.19, p-value < 0.001).

### Relationship between flood risk, human populations, and poverty

Contrary to our initial hypothesis, we uncovered an unexpected negative correlation between the event of reported floods and population density (r = -0.3, p < 0.001). Larger cities, such as Banda Aceh and Lhokseumawe, experienced fewer instances of flooding compared to smaller cities, like Aceh Utara and Singkil. This suggests that factors beyond population density may significantly influence flood susceptibility in urban areas. Our hypothesis concerning the vulnerability of impoverished communities to flooding was substantiated by our data (r = 0.24, p < 0.005; see S3 Fig). This indicates that areas with higher poverty rates tend to encounter more frequent flood events. Aceh Singkil, which witnessed an increase in the percentage of the population living below the poverty line from 17.9% in 2012 to 20.7% in 2018, endured the highest number of flooding events.

## Discussion

Protecting Aceh’s intact forests is crucial to conserving vital ecosystem services in a changing climate. Our study showed that the escalating frequency and intensity of rainfall contributed to a rising trend of reported flood events, a pattern found elsewhere in tropical Southeast Asia [60], and that this could be mitigated, to an extent, through preventing the degradation of intact forests with high tree cover. Our study contributes to highlighting the social impacts of flooding, particularly in how it hits the poorest hardest, which has broader policy implications for delivering sustainable development goals, climate change mitigation and poverty alleviation, as other studies have modelled [61, 85].

Our study found four districts (Aceh Utara, Aceh Singkil, Aceh Selatan, and Aceh Barat) experienced the majority (71%) of reported flood events between 2011 and 2018 (Fig 3). Collectively, these districts contributed 37.5% of the province’s rice production in 2019 [86], underscoring the point that flood impacts have severe negative consequences on agricultural productivity, which is critical for Aceh’s economic growth, food security and rural livelihood development. For example, a major flood that hit Aceh in 2006 damaged 8,135 ha of agricultural land and cost an estimated USD 5.5 million in Aceh Utara alone, or ~12% of the total cost to the province’s agricultural sector [34]. Conversely, Aceh Singkil is one of the main oil palm producing areas in Aceh, yet is the district with the highest proportion of people living in poverty. Oil palm cultivation was found to be a major driver of forest loss in this district and, therefore, a key contributor to the increasing frequency of flooding incidents and associated social, development and economic costs [40].

Increased flooding events associated with the conversion of forests to oil palm plantations in Aceh support findings from other studies, where local communities residing in or around oil palm plantations in Malaysia and Indonesia reported increased flood events [11, 17, 23, 87]. The conversion of tropical forests to oil palm plantation increases surface water runoff due to increased soil densities and reduces the water capacity of soils to store and regulate water flow [11, 88]. Large forest areas in Aceh Singkil have been replaced by oil palm and these lands are now increasingly threatened by more frequent flooding during the rainy season and forest fires during the dry season [89]. These flood incidents do not just affect people and infrastructure, but also lead to major losses in oil palm crop production [87, 90, 91]. For example, Abram et al. [91], found that 6.3% (15,810 ha) of oil palm in the Malaysian state of Sabah had been made commercially redundant because of palm mortality caused by flood inundation.

Protecting forest cover in all Aceh districts, but especially the most flood prone, is crucial to mitigate flooding events that could devastate the livelihoods of communities participating in agricultural activities that are also tied to poverty alleviation. Two recent studies have shown how the dual aims of forest protection and improved rural community wellbeing can be achieved in Indonesia. Findings from a conditional cash transfer program aimed at alleviating poverty in rural Sumatran communities revealed unintended benefits of reduced deforestation rates in participating villages [92], whereas villagers living in Indonesian Borneo who were highly engaged and benefiting from a rural healthcare program were more likely to conserve forest, as measured by reductions in illegal logging [93]. A recent call for a “conservation basic income”, through unconditional cash transfers averaging US$5.50 per individual per day in low-middle income countries, suggests a more cost-effective and equitable way to deliver dual conservation-livelihood goals [94]. This would be applicable to Aceh, especially when considering its special autonomy fund (since 2008), which provides an important source of revenue for rural livelihoods in Aceh, will expire in 2027, highlighting the need for alternative support.

Our study not only stresses the importance of protecting standing forests but also considers restoration options to ensure high tree cover in the critical watersheds that were identified. Spatial priority setting tools for restoration have been developed and could be applied to support provincial spatial planning processes in Aceh [95]. Restoring degraded forests in watershed areas and former timber concessions in the lowlands would contribute significantly to climate mitigation. These efforts support the achievement of provincial greenhouse gas emissions reduction targets and Indonesia’s Forestry and Other Land Use (FoLU) Net Sink 2030 goals [96]. This would provide a jurisdictional model that bucks the global trend of declining forest condition due to anthropogenic modification, which is highest in the Southeast Asia region [97].

### Study limitations

The availability of newspaper reports is an important source of information in areas with limited or no flood data. Comparison with other studies [21–23, 25] confirms the utility of extracting flood information via text mining that is combined this with spatial information. Still, we recognize several possible limitations with our approach, namely: 1) Text mining requires accurate string names (location, damage, and loss information) that are not always mentioned in news reports. To help overcome this, we used the R package ’stringdist’ [98] to enhance the precision of text extraction; 2) Underreporting of flood events may occur in sparsely populated areas [14], meaning that the absence of newspaper reports does not necessarily imply that no flooding occurred [51]. Future studies could address this limitation by combining multiple datasets, such as remote sensing data [99] and social surveys [23], to validate and complement newspaper reports; and, 3) Our online news dataset showed spatial and temporal flood event patterns that concurred with an independent dataset (from BNPB), although at a higher magnitude, but due to differences in spatial resolution between these datasets we were unable to fully validate our reported flood event dataset.

## Conclusion

Concerns over global warming and associated increases in the volume and intensity of precipitation in Southeast Asia is predicted to increase flood frequency that will cause further loss of life and infrastructure [100]. For Aceh and many other Indonesian provinces, the risk of flooding hazards are projected to increase due to climate change impacts [101], such as sea level rise [102] and land subsidence due to peatland modification [103]. In our study landscape, we identified three key factors that directly contribute to flood risk: increased precipitation, expansion of oil palm cultivation, and reduced tree cover, which are often associated with higher poverty rates. This finding underscores the critical role of maintaining intact forest to safeguard communities against flood-related events. Moreover, it emphasizes the need for evidence-based decision-making in land use and economic development planning, particularly concerning forest management. These decisions must reflect the protective benefits that forests offer against flooding and must include meaningful engagement of local communities in the planning processes [104]. Additionally, our analysis stresses the importance of forest ecosystem services at-risk areas, especially in districts with higher poverty rates, thereby providing a blueprint for lessening these predicted impacts in the future and delivering a triple win for forests and climate change, biodiversity, and poverty alleviation.

## Supporting information

S1 Text A three-step approach was employed to extract flood events using text mining. (DOCX)

S2 Text Processing tree cover (TC) and tree cover loss (TCL) from global forest cover version 1.7. (DOCX)

S1 Table Variables used in regression and correlational analyses on flood events in Aceh Province between 2011 and 2018. (DOCX)

S2 Table Comparison between binomial GLM and binomial mixed regression model with two random effects; watershed and soil type. (DOCX)

S3 Table List of models of reported flood modelling in Aceh Province, ranked based on lowest delta AICc. (DOCX)

S4 Table Comparison of regression estimates from models including individual variables (from left to right: Percentage of tree cover, percentage of oil palm, and annual rainfall). (DOCX)

S5 Table Generalized Linear Mixed Models result from top-ranked model for the relationship between reported flood events with independent variables such as percentage of tree cover (Percent TC), percentage of oil palm (Percent OP), Annual rainfall, and year as fixed effect and watershed as random effect. (DOCX)

S1 Fig Correlation plot between variables for flood study in Aceh. (TIF)

S2 Fig Residual QQ Plot from the top-ranked model. (TIF)

S3 Fig Correlation coefficient with 95% CI between reported flood event from 2011 and 2018 and population density (left) as well as percentage of people under poverty line (right) at district level (n=168) in Aceh. (TIF)
