# Supplementary material for: Tropical forest cover, oil palm plantations, and precipitation drive flooding events in Aceh, Indonesia, and hit the poorest people hardest
Source: PLoS One. 2024 Oct 14;19(10):e0311759. doi: 10.1371/journal.pone.0311759 (PMC11472921; doi:10.1371/journal.pone.0311759)
Supplement: S1 Text — (DOCX) [file pone.0311759.s001.docx]

**S1 Text. A three-step approach was employed to extract flood events using text mining**

We first extracted all Aceh district names mentioned in the articles and compared them to the corresponding district names in the Indonesian Statistics Bureau (BPS or Badan Pusat Statistik) database, which serves as the official dataset for administrative boundaries in Indonesia. Secondly, we matched the subdistrict names within each district mentioned in an article with those in the BPS database. Once we verified each district and subdistrict, we proceeded to the third step, which involved extracting the village name mentioned in the article and matching it with the BPS database. To address any inconsistencies in village names, such as variations in spelling, we conducted a similarity test between words. For example, if the similarity score between two different village names (e.g., Tapaktuan and Tapak Tuan) was high (> 0.9), we classified them as the same village. We assumed that if the names of locations at all three administrative levels (district, subdistrict, and village) matched with the BPS database, then the information for a village name was correct. Additionally, we conducted manual spot checks to test this assumption, and in all cases, the matches were correct.

The final dataset of flooding events was georeferenced by matching the administrative names, including districts, subdistricts, and villages, extracted from online news sources with the administrative boundaries for Aceh province. This matching process was performed using shapefiles provided by the Indonesian Statistics Bureau (BPS) for the year 2017. As a result, 996 village points that experienced flooding between 2011 and 2018 were identified.

To ensure the accuracy of the georeferencing, we cross-checked the administrative information obtained from the online news sources with the village location data provided by the Indonesian Geospatial Agency (Badan Informasi Geospasial), which maintains a spatial point dataset. Only flooding events that had matching location names in the media articles, BPS, and the Indonesian Geospatial Agency data were selected for inclusion in the dataset.

To handle any dissimilarities or inconsistencies between administrative names, we used a similarity index and employed the stringdist package in the R programming language [1]. This allowed us to account for variations in spelling or naming conventions.

1. Loo van der. The stringdist package for approximate string matching. R J. 2014;6: 111–122. Available: https://cran.r-project.org/package=stringdist
