# Supplementary material for: Tropical forest cover, oil palm plantations, and precipitation drive flooding events in Aceh, Indonesia, and hit the poorest people hardest
Source: PLoS One. 2024 Oct 14;19(10):e0311759. doi: 10.1371/journal.pone.0311759 (PMC11472921; doi:10.1371/journal.pone.0311759)
Supplement: S2 Text — (DOCX) [file pone.0311759.s002.docx]

**S2 Text. Processing tree cover (TC) and tree cover loss (TCL) from global forest cover version 1.7.**

Annual deforestation data were obtained from the global forest change dataset version 1.7 [1]. This dataset comprised a time-series analysis of Landsat images that characterized global forest extent and change from 2000 to 2018. The forest calculation was based on tree canopy cover from 0-100% at approximately 30 m resolution. For this study, we defined forest as any pixel with tree canopy cover ≥ 75%, which follows similar studies from the tropics [2,3]. We defined deforestation as the change in a forest pixel in 2000 to a non-forest pixel (i.e., tree canopy cover < 75%) in 2011 and subsequent years until 2018. Tree cover loss in 2011 is the loss that occurred from 2000 to 2011, and likewise for the subsequent years used in the analysis.

Before calculating tree cover loss, we applied a majority filter moving window to remove noise (known as ‘salt and pepper’) from a cell based on information from its eight neighboring cells. For each cell, an output value was generated by taking the most common value (mode) from these nine cells. After creating tree cover (TC) and tree cover loss (TCL) datasets for the years 2011 to 2018, we calculated a combination of fragmentation statistics from TC and TCL classes for each year (see S1 Table). We selected one fragmentation metric from each TC and TCL that represents the percentage of area [4]. This fragmentation statistic was chosen due to its possible relationships with floods. For instance, a watershed should have at least 30% of forest cover to maintain a sustainable water flow regulation [5]. The global forest change data were analyzed using ‘gfcanalysis’ package, while the landscape metrics analysis was carried out using ‘landscapemetrics’ package in R version 4.0.2 [6–8].

1. Hansen MC, Potapov P V, Moore R, Hancher M, Turubanova SA, Tyukavina A, et al. High-Resolution Global Maps of 21st-Century Forest Cover Change. Science (80- ). 2013;342: 850–853. doi:10.1126/science.1244693

2. Beaudrot L, Ahumada JA, O’Brien T, Alvarez-Loayza P, Boekee K, Campos-Arceiz A, et al. Standardized Assessment of Biodiversity Trends in Tropical Forest Protected Areas: The End Is Not in Sight. Dobson AP, editor. PLOS Biol. 2016;14: e1002357. doi:10.1371/journal.pbio.1002357

3. Haidir IA, Kaszta Z, Sousa LL, Lubis MI, Macdonald D., Linkie M. Felids , forest and farmland : identifying high priority conservation areas in Sumatra. Landsc Ecol. 2020;0123456789. doi:10.1007/s10980-020-01146-x

4. McGarigal K, Marks BJ. FRAGSTAT: Spatial Pattern Analysis Program for Quantifying Landscape Structure. Portland, OR: U.S. Department of Agriculture, Forest Service, Pacific Northwest Research Station; 1995.

5. Tarigan S, Wiegand K, Slamet B. Minimum forest cover required for sustainable water flow regulation of a watershed : a case study in Jambi Province , Indonesia. Hydrol Earth Syst Sci. 2018;22: 581–594.

6. Zvoleff A. gfcanalysis. In: Alex Zvoleff [Internet]. 2014. Available: http://www.azvoleff.com/gfcanalysis/

7. R Core Team. A language and environment for statistical computing. Vienna, Austria; 2017. Available: https://www.r-project.org/

8. Hesselbarth MHK, Sciaini M, With KA, Wiegand K, Nowosad J. landscapemetrics: an open-source R tool to calculate landscape metrics. Ecography (Cop). 2019; 1648–1657. doi:10.1111/ecog.04617
