# Supplementary material for: Tropical forest cover, oil palm plantations, and precipitation drive flooding events in Aceh, Indonesia, and hit the poorest people hardest
Source: PLoS One. 2024 Oct 14;19(10):e0311759. doi: 10.1371/journal.pone.0311759 (PMC11472921; doi:10.1371/journal.pone.0311759)
Supplement: S1 Table — (DOCX) [file pone.0311759.s003.docx]

**S1 Table. Variables used in regression and correlational analyses on flood events in Aceh Province between 2011 and 2018**

| No | Variable | Description | Source | Variable in 10-km grid level analysis |
| --- | --- | --- | --- | --- |
|  | **Physical and demography characteristics** | | | |
| 1 | Elevation | Elevation above sea level | Jarvis et al., 2008 | Mean elevation (m) |
| 2 | Slope | The steepness of a surface generated from elevation data | Jarvis et al., 2008 | Mean slope (degree) |
| 3 | Infrastructure | Infrastructures including road networks and river channelization at 50,000 scale | BIG, 2015 | Length of road network and river channelization s (km) |
| 4 | Annual_rainfall | Annual data of rainfall between 2011 and 2018 | Funk et al., 2015 | Mean annual rainfall (mm) |
| 5 | Percent_TC | Percentage of tree cover | Hansen et al., 2013 | Percentage of tree cover (%) in 10 km grid cell size |
| 6 | Percent_TCL | Percentage tree cover loss. | Hansen et al., 2013 | Percentage of tree cover loss (%) in 10 km |
| 7 | Percent_OP | Percentage of land under oil palm plantation | Danylo et al., 2021 | Percentage of oil palm (%) in 10 km |
| 8 | Population density | Population density in each district between 2011 and 2018 | Indonesian Statistics Bureau (BPS) of Aceh | Population density at district level |
| 9 | Percentage of poor people | Percentage of poor people in each district between 2012 and 2018. | Indonesian Statistics Bureau (BPS) of Aceh | Percentage of poor people at district level |
