# Supplementary material for: Tropical forest cover, oil palm plantations, and precipitation drive flooding events in Aceh, Indonesia, and hit the poorest people hardest
Source: PLoS One. 2024 Oct 14;19(10):e0311759. doi: 10.1371/journal.pone.0311759 (PMC11472921; doi:10.1371/journal.pone.0311759)
Supplement: S2 Table — (DOCX) [file pone.0311759.s004.docx]

**S2 Table. Comparison between binomial GLM and Binomial mixed regression model with two random effects; watershed and soil type**

|  | GLM model | | GLMM with watershed | | GLMM with soil type | |
| --- | --- | --- | --- | --- | --- | --- |
| *Covariate* | *β Est (±CI)* | *P-value* | *β Est (±CI)* | *P-value* | *β Est (±CI)* | *P-value* |
| (Intercept) | 0.03 (-0.15 – 0.20) | 0.769 | 0.24 (-0.11 – 0.59) | 0.182 | -0.49 (-0.97 – -0.01) | **0.045** |
| Annual rainfall | 0.44 (0.35 – 0.53) | **<0.001** | 0.54 (0.39 – 0.69) | **<0.001** | 0.20 (0.06 – 0.34) | **0.006** |
| Percent TC | -3.30 (-3.60 – -3.00) | **<0.001** | -3.95 (-4.32 – -3.58) | **<0.001** | -2.57 (-3.00 – -2.14) | **<0.001** |
| Random Effects | | | | | | |
| σ^2^ |  | | 3.29 | | 3.29 | |
| τ_00_ |  | | 0.96 _Watershed_ | | 0.36 _Soil_ | |
| ICC |  | | 0.23 | | 0.10 | |
| N |  | | 54 _Watershed_ | | 12 _Soil_ | |
| Observations | 4512 | | 4512 | | 4512 | |
| R^2^ Tjur | 0.128 | | 0.258 / 0.426 | | 0.144 / 0.228 | |
| Deviance | 2971.696 | | 2779.412 | | 2931.336 | |
| AIC | 2977.696 | | 2787.412 | | 2939.336 | |
| log-Likelihood | -1485.848 | | -1389.706 | | -1465.668 | |
