# Supplementary material for: Tropical forest cover, oil palm plantations, and precipitation drive flooding events in Aceh, Indonesia, and hit the poorest people hardest
Source: PLoS One. 2024 Oct 14;19(10):e0311759. doi: 10.1371/journal.pone.0311759 (PMC11472921; doi:10.1371/journal.pone.0311759)
Supplement: S3 Table — (DOCX) [file pone.0311759.s005.docx]

**S3 Table. List of models of reported flood modelling in Aceh Province, ranked based on lowest delta AICc**

| No | Variable | DAICc | AICcWt |
| --- | --- | --- | --- |
| 1 | Flood ~ Percent_TC + Percent_OP + Annual_rainfall + Year + (1\|Watershed_ID) | 0 | 1 |
| 2 | Flood ~ Percent_TC + Annual_rainfall + Year + (1\|Watershed_ID) | 13.62 | 0 |
| 3 | Flood ~ Elevation + Percent_TC + Percent_TCL + Year + (1\|Watershed_ID) | 23.34 | 0 |
| 4 | Flood ~ Percent_TC + Percent_TCL + Percent_OP + Year + (1\|Watershed_ID) | 28.96 | 0 |
| 5 | Flood ~ Elevation + Infrastructure + Percent_OP + Year + (1\|Watershed_ID) | 49.19 | 0 |
| 6 | Flood ~ Percent_TC + Year + (1\|Watershed_ID) | 58.79 | 0 |
| 7 | Flood ~ Percent_TCL + Infrastructure + Annual_rainfall + Year + (1\|Watershed_ID) | 117.03 | 0 |
| 8 | Flood ~ Slope + Year + (1\|Watershed_ID) | 120.1 | 0 |
| 9 | Flood ~ Percent_TCL + Infrastructure + Year + (1\|Watershed_ID) | 124.58 | 0 |
| 10 | Flood ~ Infrastructure + Year + (1\|Watershed_ID) | 150.41 | 0 |
| 11 | Flood ~ Elevation + Year + (1\|Watershed_ID) | 204.58 | 0 |
| 12 | Flood ~ Percent_OP + Year + (1\|Watershed_ID) | 374.51 | 0 |
| 13 | Flood ~ Percent_TCL + Year + (1\|Watershed_ID) | 414.5 | 0 |
| 14 | Flood ~ Annual_rainfall + Year + (1\|Watershed_ID) | 558.4 | 0 |
| 15 | Flood ~ 1 + (1\|Watershed_ID) | 608.91 | 0 |
