# Supplementary material for: Tropical forest cover, oil palm plantations, and precipitation drive flooding events in Aceh, Indonesia, and hit the poorest people hardest
Source: PLoS One. 2024 Oct 14;19(10):e0311759. doi: 10.1371/journal.pone.0311759 (PMC11472921; doi:10.1371/journal.pone.0311759)
Supplement: S4 Table — (DOCX) [file pone.0311759.s006.docx]

**S4 Table. Comparison of regression estimates from models including individual variables (from left to right: percentage of tree cover, percentage of oil palm, and annual rainfall).**

|  | GLMM with % TC | | GLMM with % OP | | GLMM with Annual Rainfall | |
| --- | --- | --- | --- | --- | --- | --- |
| Variables | β(±CI) | P-value | β(±CI) | P-value | β(±CI) | P-value |
| (Intercept) | 0.04 (-0.33 – 0.41) | 0.836 | -2.22 (-2.52 – -1.93) | <0.001 | -1.88 (-2.13 – -1.63) | <0.001 |
| Percent TC | -3.80 (-4.16 – -3.43) | <0.001 |  |  |  |  |
| Percent OP |  |  | 6.84 (5.86 – 7.81) | <0.001 |  |  |
| Annual rainfall |  |  |  |  | 0.26 (0.14 – 0.39) | <0.001 |
| Random Effects | | | | | | |
| σ^2^ | 3.29 | | 3.29 | | 3.29 | |
| τ_00_ | 1.26 _Watershed_ID_ | | 0.87 _Watershed_ID_ | | 0.61 _Watershed_ID_ | |
| ICC | 0.28 | | 0.21 | | 0.16 | |
| N | 54 _Watershed_ID_ | | 54 _Watershed_ID_ | | 54 _Watershed_ID_ | |
| Observations | 4512 | | 4512 | | 4512 | |
| Marginal R^2^ / Conditional R^2^ | 0.24 / 0.45 | | 0.08 / 0.27 | | 0.02 / 0.17 | |
| Deviance | 2829.267 | | 3140.952 | | 3330.986 | |
| AIC | 2835.267 | | 3146.952 | | 3336.986 | |
| log-Likelihood | -1414.633 | | -1570.476 | | -1665.493 | |
