# Supplementary material for: Tropical forest cover, oil palm plantations, and precipitation drive flooding events in Aceh, Indonesia, and hit the poorest people hardest
Source: PLoS One. 2024 Oct 14;19(10):e0311759. doi: 10.1371/journal.pone.0311759 (PMC11472921; doi:10.1371/journal.pone.0311759)
Supplement: S5 Table — (DOCX) [file pone.0311759.s007.docx]

**S5 Table. Generalized Linear Mixed Models result from top-ranked model for the relationship between reported flood events with independent variables such as Percentage of Tree Cover (Percent TC), Percentage of Oil Palm (Percent OP), Annual rainfall, and year as fixed effect and watershed as random effect.**

| Variables | Untransformed  Estimates (β) | CI | P-value |
| --- | --- | --- | --- |
| (Intercept) | -0.76 | -1.27 – -0.25 | 0.004 |
| Percent TC | -3.69 | -4.09 – -3.30 | <0.001 |
| Percent OP | 2.21 | 1.12 – 3.31 | <0.001 |
| Annual rainfall | 0.44 | 0.28 – 0.60 | <0.001 |
| Year [2012] | 0.54 | 0.08 – 1.01 | 0.022 |
| Year [2013] | 1.16 | 0.72 – 1.60 | <0.001 |
| Year [2014] | 0.81 | 0.36 – 1.26 | <0.001 |
| Year [2015] | 1.00 | 0.55 – 1.44 | <0.001 |
| Year [2016] | 0.63 | 0.18 – 1.09 | 0.006 |
| Year [2017] | 0.45 | -0.02 – 0.91 | 0.059 |
| Year [2018] | 1.05 | 0.61 – 1.49 | <0.001 |
| Random Effects | | | |
| σ^2^ | 3.29 | | |
| τ_00_ _Watershed_ID_ | 1.07 | | |
| ICC | 0.25 | | |
| N _Watershed_ID_ | 54 | | |
| Observations | 4512 | | |
| Marginal R^2^ / Conditional R^2^ | 0.28 / 0.46 | | |
| Deviance | 2718.712 | | |
| AIC | 2742.712 | | |
| log-Likelihood | -1359.356 | | |

The top ranked model shown the within-group variance (σ2), the variance of random intercepts (τ00), Intraclass Correlation Coefficient (ICC), Conditional and marginal R-squared, Deviance or likelihood ratio test, model parsimony (AIC), and log-Likelihood.
