# Supplementary figures and images for: Tropical forest cover, oil palm plantations, and precipitation drive flooding events in Aceh, Indonesia, and hit the poorest people hardest
Source: PLoS One. 2024 Oct 14;19(10):e0311759. doi: 10.1371/journal.pone.0311759 (PMC11472921; doi:10.1371/journal.pone.0311759)

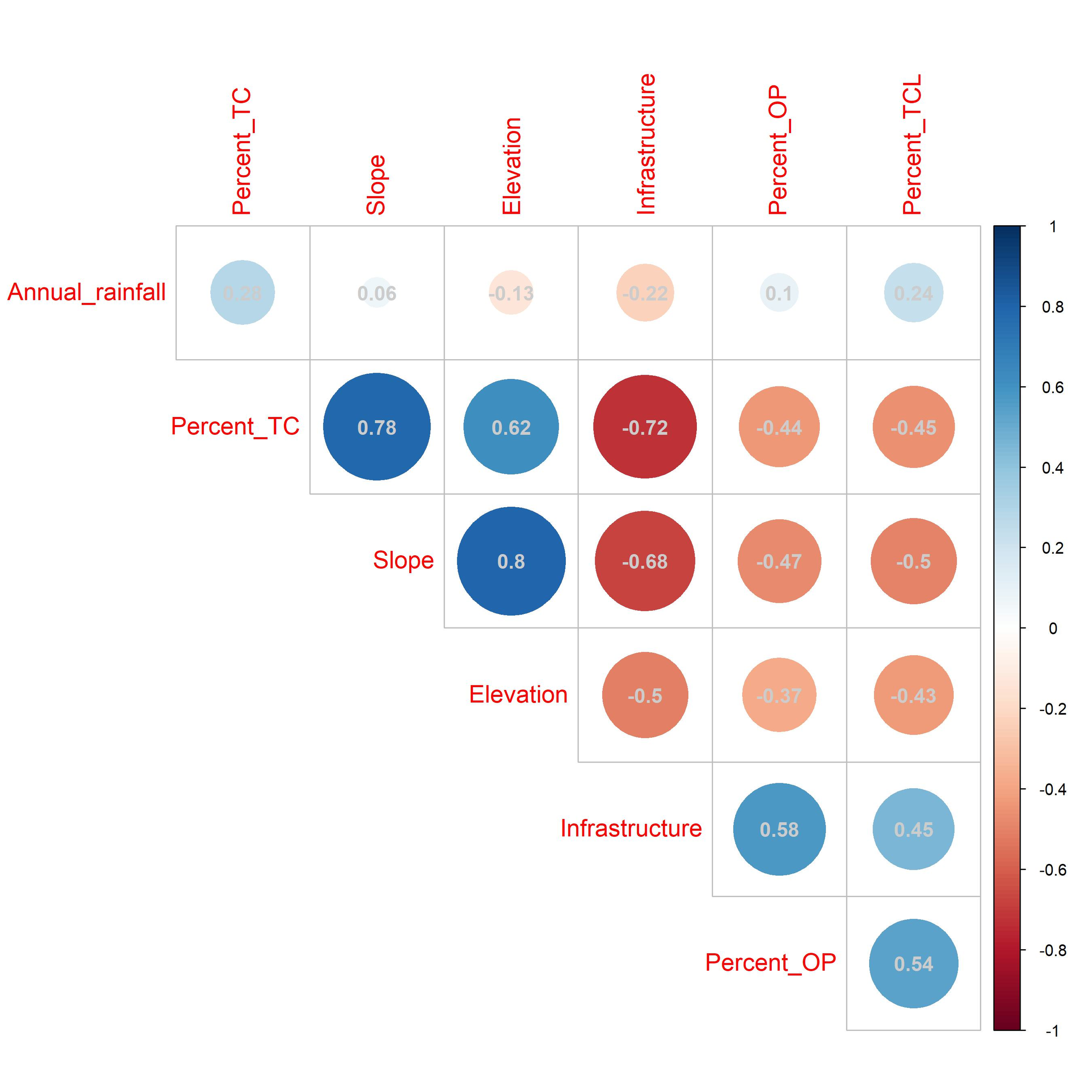

Supplement: S1 Fig — (TIF) [file pone.0311759.s008.tif]

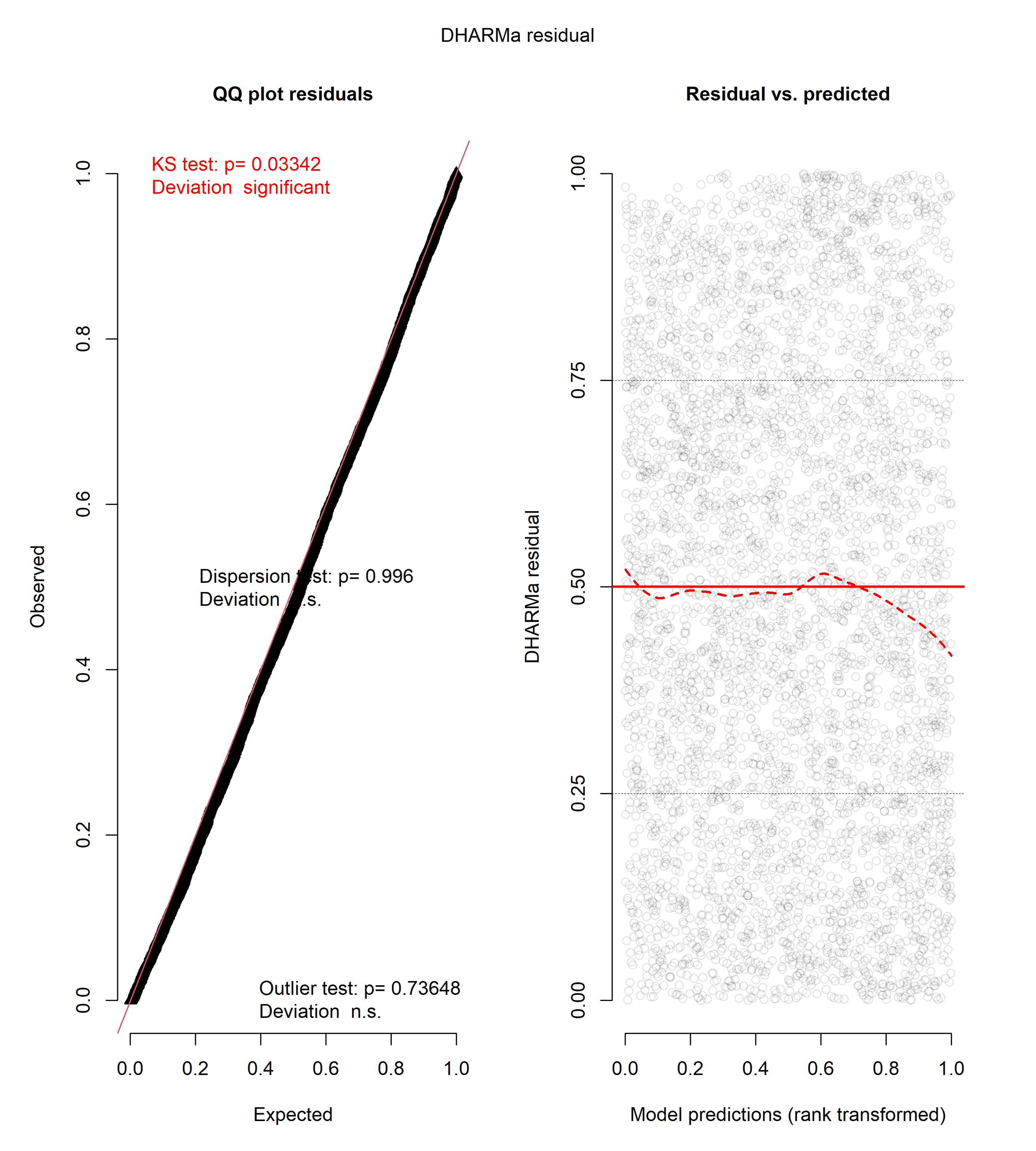

Supplement: S2 Fig — (TIF) [file pone.0311759.s009.tif]

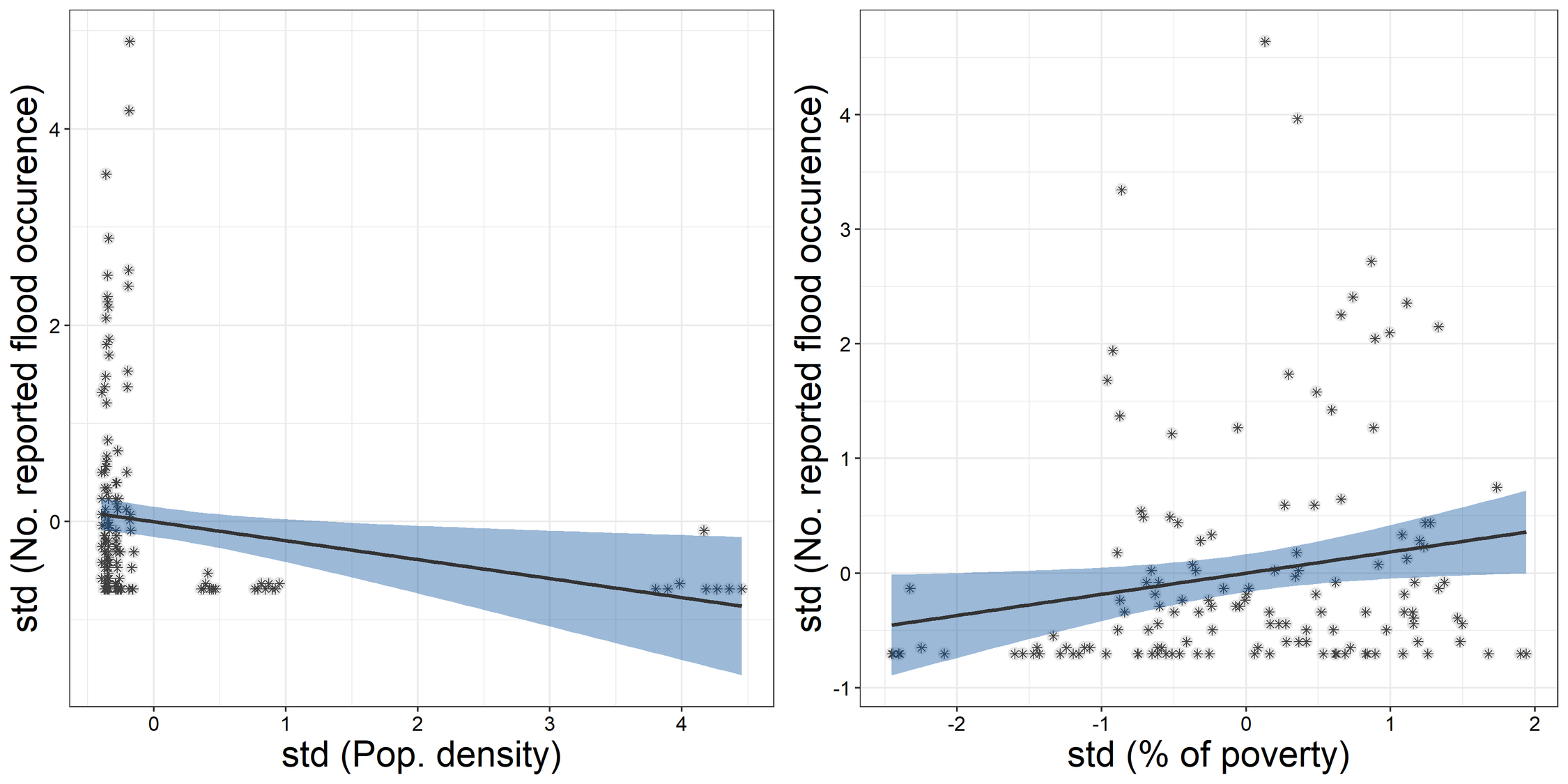

Supplement: S3 Fig — (TIF) [file pone.0311759.s010.tif]
